# Targeting the VEGF Pathway in Osteosarcoma

**DOI:** 10.3390/cells10051240

**Published:** 2021-05-18

**Authors:** Tarek Assi, Sarah Watson, Bachar Samra, Elie Rassy, Axel Le Cesne, Antoine Italiano, Olivier Mir

**Affiliations:** 1 Department of Hematology-Oncology, Faculty of Medicine, Saint Joseph University, Beirut 166830, Lebanon; tarekassi@gmail.com; 2 Department of Medical Oncology and INSERMU830, Curie Institute, 75005 Paris, France; sarah.watson@curie.fr; 3 Department of Hematology/Oncology, Presbyterian Healthcare Services, Albuquerque, NM 87110, USA; bachar.samra@gmail.com; 4 Department of Cancer Medicine, Gustave Roussy, 94800 Villejuif, France; elie.rassy@hotmail.com; 5 Sarcoma Group, Gustave Roussy, 94800 Villejuif, France; axel.lecesne@gustaveroussy.fr (A.L.C.); a.italiano@bordeaux.unicancer.fr (A.I.); 6 Department of Medical Oncology and INSERM U1218, Institut Bergonié, 33000 Bordeaux, France

**Keywords:** osteosarcoma, vascular endothelial growth factor A, protein kinase inhibitors, angiogenesis, sarcoma, bone neoplasms

## Abstract

Osteosarcoma is the most common primary tumor of the bones affecting mainly young adults. Despite the advances in the field of systemic anticancer therapy, the prognosis of relapsed of metastatic osteosarcoma patients remain dismal with very short survival. However, the better understanding of the pathophysiology of this subtype of sarcoma has led to the identification of new targeted agents with significant activity. In fact, increased angiogenesis plays a major role in the tumor growth and survival of osteosarcoma patients. Several targeted agents have demonstrated a significant anti-tumor activity including multi-kinase inhibitors. In this review, we will discuss the pathophysiology, rationale, and role of targeting angiogenesis via the VEGF pathway in patients with osteosarcoma with emphasis on the published clinical trials and future directions.

## 1. Introduction

High-grade malignant osteosarcoma is a rare tumor with a worldwide incidence of 3–4 cases per million [1]. It is the most common primary tumor of the bones, affecting mainly young adults with a peak incidence in the second decade of their life [1]. These tumors are characterized by the presence of malignant mesenchymal cells and increased osteoid production. They are classified according to multiple distinct histological subtypes (conventional or osteoblastic—the most common subtype—or chondroblastic, fibroblastic, telangiectatic, small cell, surface, and secondary) [1]. Osteosarcoma are locally invasive with a high tendency to produce distant metastases, mainly to the lungs [2]. Although approximately 80% of patients present with localized disease, approximately one third of patients will eventually exhibit disease recurrence or metastases despite optimal local therapy and neoadjuvant/adjuvant chemotherapy [3,4].

Despite the dramatic therapeutic advancements in the field of systemic anticancer treatments over the past three decades, the prognosis of patients with advanced/metastatic osteosarcoma remains poor with less than 20% of long term survivors and limited therapeutic options. Nevertheless, 30–40% of patients with oligometastatic lung disease may potentially be cured with multimodality therapy through improvement of surgical approaches and multi-agent chemotherapy [5,6]. Predictors for better outcome include site and number of metastases (bone metastases being associated with worse outcome compared with lung metastases) and resectability of metastatic disease. The optimal chemotherapy regimen for metastatic osteosarcoma has not been well defined, but the most widely used regimen for treatment-naïve patients is the combination of consists of high-dose methotrexate, doxorubicin, and cisplatin (MAP) with or without ifosfamide [7].

Nevertheless, relapsing and/or metastatic osteosarcoma patients have a dismal prognosis with a median overall survival of less than 8 months, without any new drug approved in this setting for the past 30 years [8,9,10]. Current plausible therapeutic options in this setting beyond the first line have limited efficacy and consist of conventional chemotherapy either as single agents or in doublet-combinations [11]. Chemotherapy combinations have demonstrated limited efficacy in the advanced lines of treatment with response rates ranging between 3 and 29%, and a median PFS of less than 4 months [12,13,14].

Recently, the advent of immune checkpoint inhibitors has drastically changed the management of a multitude of solid and hematological tumors, with significant impacts on survival and prognosis [15,16,17,18]. Unfortunately, despite high PD-L1 expression levels (ranging from 14 to 75%) and promising results in preclinical models [19,20], immune checkpoint inhibitors in unselected patients with relapsed osteosarcoma have shown very limited clinical activity, with an overall response rate (ORR) of <10% (e.g., 4.5% with pembrolizumab in the SARC028 trial [21] and 6.7% with pembrolizumab plus metronomic cyclophosphamide in the PEMBROSARC trial) [22].

Nevertheless, a better understanding of the pathophysiology of osteosarcoma has led to the identification of new potential therapeutic targets with the pivotal role of increased angiogenesis in both tumor growth and metastatic progression. Several targeted agents have shown significant anti-tumor activity, including multi-kinase inhibitors (MKI) [23]. In this review, the pathophysiology, rationale and role of targeting angiogenesis via the vascular endothelial growth factor (VEGF) pathway in osteosarcoma will be discussed. A comprehensive review of the literature was carried out by searching PubMed database and the bibliography sections of relevant publications published in English between 1 January 2000 and 31 January 2021. This review summarizes the current clinical evidence and future directions in this setting.

## 2. Clinical Evidence

### 2.1. Role of VEGF in Osteosarcoma

**Figure 1:**
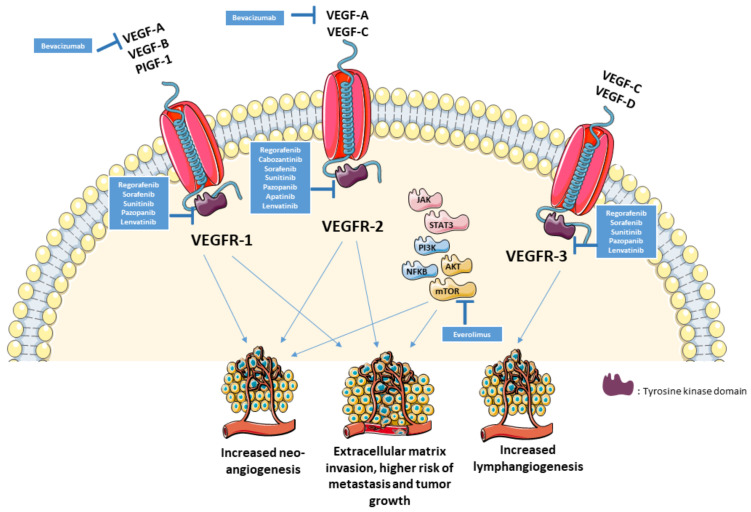
VEGF pathway in osteosarcoma and potential therapeutic targets.

Angiogenesis is one of the six essential hallmarks of tumorigenesis by impacting tumor growth, and metastatic potential [24]. VEGF, a crucial factor in the angiogenesis and vasculogenesis, mainly acts on different cell types mainly on endothelial cells. It plays a major role in the physiologic vascular homeostasis of various tissues but also in the molecular pathogenesis of metastasis and tumor growth [25]. The expression of VEGF-A (by immunohistochemistry) in osteosarcoma has been associated with a higher risk of lung metastasis, and poorer survival [26,27,28]. Additionally, *VEGFA* gene amplification has been shown to be a poor prognostic factor for tumor-free survival [26]. These findings have generated interest in drugs targeting VEGF pathway in order to improve outcomes. The inhibition of VEGF signaling halts cell growth and stimulates apoptosis in osteosarcoma cells [29]. VEGFR2 (vascular endothelial growth factor receptor 2), the main VEGF-A receptor involved in angiogenesis and vasculogenesis, and PD-L1, expressed in 64.5% and 35.5% of osteosarcoma cells, respectively, were associated with a pro-metastatic effect in the lungs, and tumor growth [30]. There was also a significant correlation between PD-L1 and VEGFR2 expression in osteosarcoma (*p* = 0.0009) while both had negative impact on survival [30]. Figure 1 illustrates the role of VEGF in osteosarcoma and the potential therapeutic targets.

Osteosarcoma is a genetically unstable tumor with no specific pattern of cytogenetic characteristics, and profound interpatient heterogeneity [31]. Osteosarcoma pathogenesis may involve genetic aberrations of the VEGF, mTOR, and Wnt (Wingless-related integration site) signaling pathways; inactivation of tumor suppressors p53 and Rb (retinoblastoma); and amplification of *APEX1 (Apurinic/Apyrimidinic Endodeoxyribonuclease 1)*, *MYC*, *CCN1 (Cellular Communication Network Factor 1)*, *RAD21* (RAD21 *cohesin complex component)*, *AURKB (Aurora Kinase B)* and *CDK4 (cyclin-dependant kinase 4)*, *RECQL4 (RecQ Like Helicase 4)*, *RPL8 (Ribosomal Protein L8)*, *HDMX (human homologue of Mdm2 (mouse double minute 2) proto-oncogene)*, and *VEGFA* [32,33]. Targeting these patient-specific SCNAs (somatic copy-number alterations) may lead a to a control of tumor growth suggesting a role for genome-matched personalized therapy. Constitutional activation or wild-type MET (a transmembrane tyrosine kinase receptor with a significant role in proliferation, survival, and motility), the receptor for HGF (Hepatocyte Growth Factor) involved in endothelial cell migration, plays a role in the transformation of primary human osteoblasts into osteosarcoma cells; the introduction of dominant-negative MET into osteosarcoma cells reduces in vivo tumorigenesis and transformation [34].

Up to 70% of osteosarcoma have a loss-of-function mutation in the tumor suppressor gene encoding the Rb-associated protein [35]. Application of gene microarrays has shown an upregulation of genes affecting the extracellular matrix (ECM) that are involved in adhesion, cell and leukocyte migration suggesting a role of ECM dysregulation in OST tumorigenesis [36]. Furthermore, genomic sequencing of 66 pediatric and adult osteosarcoma using MSK-Impact, a large Next Generation sequencing (NGS) assay has identified at least one targetable molecular alteration in 14 pts (21%) including amplification of *CDK4* and/or *MDM2* (14% each) [37]. The most common frequent copy-number alterations was the amplification at the 6p12–21, involving *VEGFA* (27%) and often *CCND3* [37]. Up to 40% of tumors had platelet-derived growth factor receptor A (PDGFRA) or *VEGFA* amplifications, thus suggesting an interest of anti-angiogenic agents in this entity [37].

### 2.2. Clinical Evidence with Anti-VEGF(R) Agents

#### 2.2.1. Regorafenib

Regorafenib is an oral MKI affecting vasculature and tumor microenvironment with targeting of specific kinase proteins (VEGFR1,2,3, PDGFR, FGFR, KIT, BRAF, and RET) [38]. It is approved by the Food and Drug Administration (FDA) for the management of advanced gastrointestinal stromal tumors, colorectal, and hepatocellular carcinoma [39,40]. First, a phase 1 trial in advanced solid tumors had demonstrated encouraging single agent activity in an osteosarcoma patient [41]. Second, regorafenib has demonstrated activity in non-adipocytic soft tissue sarcoma, with a significant prolongation of progression-free survival (PFS) [42].

The role of regorafenib in relapsed OST was established by two randomized phase 2 trials [43,44]. The REGOBONE trial was a phase 2, randomized, double-blind, placebo-controlled study that included 38 metastatic osteosarcoma patients. Patients were randomized on a 2:1 basis to receive either regorafenib (160 mg daily for 21 days every 28 days) or a placebo, after failure of one or 2 lines of therapy. Crossover was allowed upon progression. The PFS at 8 weeks was 65% in the experimental arm versus 0% in the placebo group. The median PFS and overall survival (OS) were 16.4 weeks and 11.3 months in the regorafenib arm versus 4.1 weeks and 5.9 months in the placebo arm, respectively. Serious adverse events were more common in the experimental arm (24% vs. 0%), with the most common grade ≥ 3 adverse events being hypertension (24% vs. 0%) and hand–foot skin reaction (10% vs. 0%) [43]. Similar activity was demonstrated in the SARC024 trial, a phase 2 study evaluating the role of regorafenib in specific subtypes of sarcomas. A total of 42 patients were randomized between regorafenib (160 mg daily for 21 days every 28 days) or a placebo (with a possible crossover upon progression). The study evidenced a significant improvement in PFS (3.6 vs. 1.7 months; HR = 0.42 CI 95% (0.21–0.85); *p* = 0.017); there was no significant difference in terms of OS (11.1 vs. 13.4 months; HR = 1.26 CI 95% (0.61–3.13); *p* = 0.62). Three patients had partial responses (ORR (overall response rates) = 13%). The most common toxicities (grade ≥ 3) were: hypertension (14%), thrombocytopenia, hypophosphatemia, maculopapular rash, and extremity pain (9% each) while one patient had grade 4 colonic perforation [44]. According to the NCCN guidelines, regorafenib is considered a category 1 option in the management of relapsed/refractory or metastatic osteosarcoma patients [45].

Regorafenib is being currently evaluated as a maintenance treatment after a first line of chemotherapy in relapsed osteosarcoma in a randomized, placebo-controlled phase 2 study (NCT04055220).

#### 2.2.2. Cabozantinib

Cabozantinib, a MKI targeting VEGFR2 and MET, is an FDA approved agent for the treatment of renal cell carcinoma and medullary thyroid carcinoma [46]. Cabozantinib exerts anticancer activity primarily through receptor kinase inhibition of tumor cabozantinib demonstrated an in vitro and in vivo activity in osteosarcoma tumor models [47]. Through the inhibition of the ERK and AKT signaling pathways, cabozantinib may lead to a decrease in the proliferation and migration of osteosarcoma cells, and a decrease in the production of RANK ligands by osteoblasts [48]. Furthermore, through the inhibition of VEGFR2 and c-MET, cabozantinib modulates the expression of osteoclast/osteoblast marker genes including *Receptor Activator of Nuclear Factor (RANK)*. RANK is expressed on osteosarcoma cells and is also produced by osteoblasts in the bone microenvironment, which could lead to a pro-tumorigenic effect in osteosarcoma cells expressing RANK [49,50]. Cabozantinib has also successfully reduced the production of osteoprotegerin, a soluble receptor of RANK ligand, in human osteoblasts, thus confirming its significant impact on the bone microenvironment [51]. Moreover, c-MET is overexpressed in osteoblasts, contributing to their transformation into osteosarcoma cells [34,52]. The inhibition of c-MET with crizotinib induced significant reduction in the malignant potential of osteosarcoma cells in in vivo and in vitro models [53,54]. Additionally, HGF (the only known ligand for c-MET) has been implicated in the resistance to VEGFR inhibitors like sunitinib, thus providing a rationale for the use of cabozantinib to overcome resistance to other VEGFR inhibitors [55].

Cabozantinib has demonstrated a meaningful clinical activity in Ewing sarcoma and osteosarcoma patients with an acceptable toxicity profile [56]. In the CABONE trial, a multicentric single-arm phase 2 trial, 90 patients (12 years and older) with recurrent or metastatic Ewing sarcoma or OST received cabozantinib (60 mg orally for a cycle of 28 days or 40 mg/m^2^ in <16 years-old) until progression or toxicity. In the osteosarcoma cohort (42 evaluable patients), the ORR was 12% (n = 5) while PFS at 6 months was 52%. The median PFS was 6.7 months and the median OS reached 10.6 months. The most common severe toxicities (grade ≥ 3) were hypophosphatemia, elevated aspartate aminotransferase, palmar-plantar syndrome, and neutropenia. Among patients with osteosarcoma, a low VEGF-A concentration (<12.5 pg/mL) was associated with a better OS (13.2 vs. 8.2 months, *p* = 0.014) while high soluble MET levels (>300.6 ng/mL) were associated with a better PFS (7.8 vs. 5.4 months, *p* = 0.016).

#### 2.2.3. Sorafenib (Alone or in Combination with mTOR Inhibitors)

Sorafenib is an oral agent targeting Kit, RAF, VEGFR1,2,3 and PDGFRA, with FDA approval in renal cell carcinoma, differentiated thyroid carcinoma and hepatocellular carcinoma [57,58,59]. Sorafenib has shown clinical activity in osteosarcoma, both as single agent and in combination with mammalian target of rapamycin (mTOR) inhibitors. In fact, mTOR is an essential serine/threonine kinase which acts as a downstream mediator in the PI3K pathway, thus playing an important role in the regulation of cell functions (survival, cell growth and angiogenesis) [60,61].

Grignani et al. [62] evaluated the activity of sorafenib (400 mg twice daily, until progression) in a multicentric non-randomized phase 2 trial on 35 patients with unresectable high grade osteosarcoma. The 4-months PFS was 46%, and the median PFS and OS were 4 and 7 months, respectively. The ORR was 14% with three partial responses. The most common reported side-effects grade were hand foot reaction, anemia, and thrombocytopenia [62]. Interestingly, sorafenib led to a reduction in ^18^F-fluorodeoxyglucose PET uptake and tumor density.

While sorafenib inhibits the activity of the mTORC1 complex, an alternative effect was exhibited on mTORC2 complex with the activation and promotion of tumor growth [63]. mTORC1 (mammalian target of rapamycin complex I) and mTORC2 are central regulators of cellular growth and their hyperactivation is involved in the pathogenesis of several diseases including cancer [64,65]. Through the inhibition of both mTORC1 and mTORC2, the combination of sorafenib with everolimus potentiated the anti-angiogenic effect, increased the anti-proliferative and pro-apoptotic effect, and reduced the metastatic potential [63]. The addition of sirolimus to sorafenib had an additive effect on enhancing the antiproliferative, pro-apoptotic, and antiangiogenic effect with a reduction in tumor growth and metastasis propensity by their effect on both mTORC1 and mTORC2 in OST cell lines using mouse models [63].

In a non-randomized phase 2 study by Grignani et al. [66], 38 adult patients with unresectable relapsed osteosarcoma received the combination of sorafenib (800 mg daily) and everolimus (5 mg daily) until progression or intolerable toxicity. Seventeen out of 38 patients (45%) were progression-free at 6 months, while the median PFS was 6 months. The ORR was 10% (n = 4) and the disease control rate was 63% (24 of 38 patients). Additionally, 30% of patients (n = 10) had non-dimensional responses on ^18^FDG PET-scan. Immunohistochemical staining with P-ERK1/2 and P-RPS6 were associated with better PFS at 6 months. The most common side effects (grade ≥ 3) were lymphopenia, hypophosphatemia, and hand–foot syndrome. Based on aforementioned data, NCCN lists sorafenib as an acceptable option both as monotherapy and in combination with everolimus for OST [45].

#### 2.2.4. mTOR Inhibitors

The overexpression of mTOR in osteosarcoma is associated with higher risk of progression and poorer survival [67]. The complexity of the PI3K/AKT/mTOR pathways and the multiple mechanisms of resistance related to the activation of this pathway, have urged researchers to assess the role of mTOR inhibitors in combination with various drug agents to overcome the resistance. Building upon their synergistic activity with anti-VEGFR MKIs, mTOR inhibitors have been combined with cytotoxic chemotherapy.

A phase 1 study of the combination of gemcitabine and sirolimus led to the successful inhibition of mTOR and demonstrated encouraging antitumor activity [68]. In a single-arm phase 2 trial, 35 patients with recurrent or advanced osteosarcoma received gemcitabine (800 mg/m^2^ i.v. on days 1 and 8 of a 21-day cycle) and rapamycin 5 mg orally daily. The ORR was 6%, including 2 partial responses. The median PFS was 2.3 months; the PFS at 4 months was 44%. The median OS was 7.1 months. The most common side effects grade were cytopenia and fatigue. Notably, the expression of P-ERK1/2 correlated with superior OS but not PFS, while the expression of RRM1 correlated with an inferior PFS and OS [69].

In a “real world” retrospective analysis in 29 patients with osteosarcoma treated most commonly with sirolimus plus oral cyclophosphamide, disease stabilization was reported in 45.5% of patients, with a median duration of response of 4.8 months. The median PFS was 3 months. The median OS at one year reached 30% with sirolimus [70].

#### 2.2.5. Pazopanib

Pazopanib is a MKI that targets different kinases including VEGFR1,2,3, PDGFRA, and PDGFRB. It is approved by the FDA for the treatment of non-adipocytic soft tissue sarcomas after failure of standard chemotherapy, based on the results of the phase 3 PALETTE trial that demonstrated improved PFS over a placebo [71]. Preclinical mouse models using osteosarcoma cells showed that pazopanib led to the disruption of the vascular barrier and inhibited the trans-endothelial migration of tumor cells [72]. Clinical activity of pazopanib were reported in 3 patients with advanced/recurrent osteosarcoma [73]. Longhi et al. reported on 9 patients with recurrent/metastatic osteosarcoma receiving pazopanib 800 mg daily after at least 2 lines of therapy. The ORR was 37.5% (with 3 partial responses) and the disease control rate was 75%. The most common side effects grade were hand–foot syndrome, hypertension and thrombocytopenia (2 patients [25%] each) [74]. In another series, 15 patients received pazopanib (13 patients at the dose of 800 mg and 2 patients at 400 mg), resulting in median PFS and OS of 6 and 7 months, respectively. The disease control rate was 60% (9 out of 15 patients) with only one patient with confirmed partial response. Hypertension and thrombocytopenia were the most common adverse events (20% each) [75].

A phase 2 trial combined pazopanib (800 mg daily) with topotecan (8 mg on days 1, 8 and 15) on a 28-days cycles in three different cohorts of STS and bone sarcomas. In the osteosarcoma cohort (n = 17), the ORR was 6% with a clinical benefit rate of 88%. The median PFS at 3 months was 62.5%, and median PFS and OS were 4.5 and 11.1 months, respectively [76].

#### 2.2.6. Other Agents

Apatinib, another MKI that targets VEGFR2, has been used for several years with an off-label indication in advanced sarcomas with discordant results [77,78]. In in vivo studies, apatinib successfully inhibited the invasion and migration of osteosarcoma cells through suppression of epithelial–mesenchymal transition (EMT) and inactivation of signal transducer and activator of transcription 3 (STAT3), which is involved in cell growth through downstream signaling molecules (BCL2 (B-cell lymphoma 2) and cyclin D1). Apatinib plays an important role in osteosarcoma cell growth suppression in in vivo models [79]. It reduces the PD-L1 expression thus demonstrating an active role in the immune escape suppression, in addition to its angiogenic effects [30]. Moreover, it induces autophagy, cell cycle arrest, and apoptosis through deactivation of VEGFR2/STAT3/BCL2 signal pathway. The role of apatinib in osteosarcoma was assessed in a non-randomized phase 2 trial on 37 patients, at a dose of 500 mg if BSA (body surface area) < 1.5 m^2^, or 750 mg if BSA ≥ 1.5 m^2^). The ORR was 43.2% and the median PFS and OS were 4.5 and 9.9 months, respectively. The most common grade ≥ 3 side effects were pneumothorax (16.2%), palmo-plantar erythrodysesthesia syndrome (8.1%), wound dehiscence (10.8%), proteinuria (8.1%), and diarrhea (8.1%) [80].

Lenvatinib, a MKI targeting VEGFR1,2,3 and FGFR, was assessed at the dose of 14 mg/m^2^ alone or at the dose of 11mg/m^2^ in combination with ifosfamide 3 g/m^2^ and etoposide 100 mg/m^2^ i.v. days 1–3. Among 16 young adults with recurrent or refractory osteosarcoma, the disease-control rate was 50% (including one partial response) and the 4-month PFS rate was 33%. The most common side effects were hypothyroidism, proteinuria, and diarrhea [81].

Sunitinib, a MKI targeting VEGFR1,2,3, Kit, PDGFRA/B, FLT-3 and RET has also shown efficacy in the reduction of the primary tumor proliferation and the tumor vasculature in cell-derived osteosarcoma mouse models [82].

Finally, the association of bevacizumab (a monoclonal antibody to VEGF-A) given at the dose of 10mg/kg every 14 days with everolimus 10 mg daily +/− erlotinib was investigated in a phase 1 trial in advanced solid tumors. One partial response and one prolonged stable disease (over 20 months) were observed in heavily pretreated osteosarcoma patients [83].

Table 1 includes all published data on the role of anti-VEGF agents in relapsed or metastatic osteosarcoma patients.

## 3. Conclusions

Despite the recent improvements in systemic anticancer treatments, the prognosis of relapsing osteosarcoma patients remains dismal. Nevertheless, the proper identification of factors involved in the increased angiogenic activity and tumorigenesis of osteosarcoma have paved the way for a new therapeutic pathways. Several anti-angiogenic MKIs have demonstrated a significant activity in osteosarcoma with prolonged disease control. Ongoing clinical trials (listed in Table 2) will help identifying emerging candidates for the drug armamentarium of relapsed osteosarcoma.

Notably, the benefit of anti-VEGF agents in osteosarcoma appears to be modest and transient, suggesting a role for earlier introduction (as maintenance therapy after a first line of treatment for instance), or combination with other drug classes (when allowed by the toxicity profile of each drug). Mechanisms of clonal resistance are an active area of research and may provide a rationale for potential therapeutic strategies. Future trials should eagerly integrate these agents, aiming to optimize the outcomes of patients with advanced osteosarcoma.

## Figures and Tables

**Table 1:** Clinical data on the use of anti-VEGF agents in osteosarcoma patients.

Author	N (n = OST)	Phase	Type of Study	Drug	Control	Population	ORR	PFS (Months)	OS (Months)	Side Effects (Grade 3–4)	Additional Survival Data
Duffaud et al. (2019) [43]	38 (26)	2	Non-comparative, double blind, prospective, randomized	Regorafenib (160 mg daily for 21 days q28 days)	Placebo	Metastatic OST (10 years or older) after failure of 2 lines of therapy	8% vs. 0	16.4 vs. 4.1 weeks	11.3 vs. 5.9	Hypertension (24% vs. 0%) and hand-foot reaction (10% vs. 0%)	PFS at 8 weeks (65% vs. 0)
Davis et al. (2019) [44]	42	2	Double blind, prospective, randomized	Regorafenib (160 mg daily for 21 days q28 days)	Placebo	Metastatic OST (10 years or older) after failure of 1 lines of therapy	13.6 vs. 0	3.6 vs. 1.7	11.1 vs. 13.4	hypertension (14%) followed by thrombocytopenia, hypophosphatemia, maculopapular rash and extremity pain (9% each)	PFS at 8 weeks (79 vs. 25)
Italiano et al. (2020) [56]	90 (45)	2	Prospective, single arm	Cabozantinib (60 mg orally for a cycles of 28 days or 40 mg/m^2^ in <16 y)	NA	Recurrent or metastatic OST and Ewing sarcoma (10 years or older)	12	6.7	10.6	hypophosphatemia, elevated aspartate aminotransferase, palmar-plantar syndrome and neutropenia	6-month non-progression = 33%
Grignani et al. (2012) [62]	35	2	Prospective, single arm	Sorafenib 400 mg twice daily until progression	NA	Relapsed or unresectable OST (>14 years) after standard therapy	8	4	7	anemia, thrombocytopenia (6%)	PFS at 4 months = 46%
Grignani et al. (2015) [66]	38	2	Prospective, single arm	Sorafenib 800 mg + everolimus 5 mg daily	NA	Relapsed or unresectable OST after standard therapy	10	5	11	lymphopenia, hypophosphatemia and hand–foot syndrome	PFS at 6 months = 45%
Martin-Broto et al. (2017) [69]	35	2	Prospective, single arm	Gemcitabine (800 mg/m^2^ on day 1 and 8 on a 21-day cycle) and rapamycin 5 mg daily	NA	Relapsed or unresectable OST after standard therapy	6%	2.3	7.1	cytopenia and fatigue	PFS at 4 months = 44%
Penel-Page et al. (2015) [70]	23 (18 combo)	NA	Retrospective	Sirolimus ± cyclophosphamide	NA	Relapsed OST after standard therapy	13	3	NA		PFS at 4 months = 40%
Longhi et al. (2018) [75]	15	NA	Retrospective	Pazopanib 800 mg daily	NA	Metastatic or unresectable OST after standard therapy	7	6	7	Hypertension and thrombocytopenia (20% each)	
Agulnik et al. (2018) [76]	139 (17)	2	Prospective, single arm	Pazopanib (800 mg daily) with topotecan (8 mg on day 1, 8 and 15) on a 28-days cycles	NA	Metastatic or unresectable OST after standard therapy	6	4.5	11.1	In all population: neutropenia (42), thrombocytopenia (29), hypertension (16) and anemia (12)	PFS at 3 months = 62.5%
Xie et al. (2019) [80]	37	2	Prospective, single arm	Apatinib (500 mg (body surface area) <1.5, or 750 mg if BSA ≥ 1.5)	NA	Relapsed or unresectable OST after standard therapy	43.24	4.5	9.87	pneumothorax (16.2%), palmo-plantar erythrodysesthesia syndrome (8.1%) wound dehiscence (10.8%), proteinuria (8.1%) and diarrhea (8.1%)	PFS at 4 months = 57%
Gaspar et al. (2018) [81]	16 (P.2) and 7 (1b)	1b–2	Prospective, single arm	Lenvatinib 14 mg/m^2^ (P.2) or 11 mg/m^2^ in combination with ifosfamide 3 g/m^2^ and etoposide 100 mg/m^2^ days 1-3 (P.1b)	NA	Relapsed or unresectable OST after standard therapy	6.25 (P.2) and 14.2 (P.1b)	NA	NA	Back pain and dyspnea (12.5% each)	

PFS: progression-free survival; OS: overall survival; NA: not applicable; OST: osteosarcoma; ORR: overall response rate.

**Table 2:** Ongoing clinical trials of anti-VEGF agents in osteosarcoma.

Clinicaltrials.gov Identifier	Phase	N	Title	Clinical Setting	Type of tumors	Interventional Arm	Control Arm	Primary Endpoint	Start Date	End Date	Status
NCT04154189	2	72	A Multicenter, Open-label, Randomized Phase 2 Study to Compare the Efficacy and Safety of Lenvatinib in Combination with Ifosfamide and Etoposide Versus Ifosfamide and Etoposide in Children, Adolescents and Young Adults with Relapsed or Refractory Osteosarcoma (OLIE)	Children, Adolescents, and Young Adults with Relapsed or Refractory Osteosarcoma	Osteosarcoma	Lenvatinib 14 mg/m^2^ d1–21 + Ifosfamide 2 g/m^2^ D1–3 + Etoposide 100 mg/m^2^ D1–3 for 5 cycles	Ifosfamide 2 g/m^2^ D1–3 + Etoposide 100 mg/m^2^ D1–3 for 5 cycles	PFS at 4 months	March 2020	December 2022	Recruiting
NCT03900793	1	41	A Phase I/Ib Study of Losartan in Combination with Sunitinib in the Treatment of Pediatric and Adult Patients with Relapsed or Refractory Osteosarcoma	Pediatric and Adult Patients with Relapsed or Refractory Osteosarcoma	Osteosarcoma	Losartan + Sunitinib	NA	DLT + Phase 2 dosing	August 2019	February 2025	Recruiting
NCT04055220	NA	168	A Randomized, Placebo-controlled, Double-blinded, Multicentre Study Evaluating the Efficacy and Safety of Regorafenib as Maintenance Therapy After First-line Treatment in Patients with Bone Sarcomas	Maintenance Therapy After First-line Treatment in Patients with Bone Sarcomas	Osteosarcoma + Bone sarcomas	Regorafenib 120 D1–21 for a 28-day cycles for 13 cycles	Placebo	Relapse free survival	March 2020	October 2024	Recruiting
NCT03742193	2	43	A Phase II Study of Gemcitabine-docetaxel Chemotherapy with VEGFR Inhibitor (Apatinib) for Pulmonary Resectable Metastases of Osteosarcoma	Second line in patients with resectable lung metastasis	Osteosarcoma	Apatinib 250 mg bid + Gemcitabine 900 mg/m^2^ on D1 and D8 + Docetaxel 75 mg/m^2^ on 21 day cycles for 7–8 cycles with maintenance apatinb (before and after surgery)	NA	PFS at 12 months	March 2019	September 2022	Recruiting
NCT03277924	1 and 2	270	Phase I–II Trial of Sunitinib Plus Nivolumab After Standard Treatment in Advanced Soft Tissue and Bone Sarcomas	Metastatic or relapsing bone sarcomas	Osteosarcoma + Bone sarcomas	Sunitinib 37.5 mg continuously + Nivolumab 240 mg every 2 weeks	NA	PFS at 6 months	May 2017	September 2022	Recruiting
NCT03359018	2	43	Apatinib Mesylate Plus Anti-PD1 Therapy (SHR-1210) in Locally Advanced, Unresectable or Metastatic Osteosarcoma(APFAO)Refractory to Chemotherapy: a Single Institution, Open-label, Phase 2 Trial	Locally Advanced, Unresectable or Metastatic Osteosarcoma(APFAO)Refractory to Chemotherapy	Osteosarcoma	Apatinb 500 mg or 250 mg daily + SHR-1210 3 mg/kg every 2 weeks until progression	NA	PFS and CBR	January 2018	January 2020	Completed
NCT04044378	1 and 2	80	Famitinib Malate (SHR1020) Plus Camrelizumab (SHR 1210) Versus Famitinib Malate Alone Versus Famitinib Malate Plus Ifosfamide Locally Advanced, Unresectable or Metastatic Osteosarcoma Progression Upon Chemotherapy: A Phase Ib/II Randomized and Controlled Dose-Escalation Trial	Locally Advanced, Unresectable or Metastatic Osteosarcoma(APFAO)Refractory to Chemotherapy	Osteosarcoma	Famitinib (escalation dose) then Famitinib 20 mg daily (phase 2) + Camrelizumab 200 mg every 2 weeks/Famitinib + Ifosfamide 3 g/m^2^ D1–3 and D 15–17 of 28-day cycles for 5 cycles	Famitinib 20 mg daily (phase 2)	ORR and PFS	August 2019	September 2022	Recruiting
NCT02389244	2	132	A Randomized Phase II, Placebo-controlled, Multicenter Study Evaluating Efficacy and Safety of Regorafenib in Patients with Metastatic Bone Sarcomas	Relapsing metastatic	Osteosarcoma + Bone sarcomas	Regorafenib 160 (or 82 mg/m^2^ in pediatric) D1–21 for a 28-day	Placebo	PFS	September 2014	March 2023	Recruiting
NCT02357810	2	136	A Phase II Study of Pazopanib With Oral Topotecan in Patients with Metastatic and Non-resectable Soft Tissue and Bone Sarcomas	Relapsing metastatic	STS + Bone sarcomas	Pazopanib D1–D28 + Topotecan D1,8,15	NA	PFS at 12 weeks	February 2015	June 2022	Recruiting
NCT02048371	2	150	SARC024: A Blanket Protocol to Study Oral Regorafenib in Patients with Selected Sarcoma Subtypes	Relapsing metastatic	Selected STS including osteosarcoma	Regorafenib 160 D1–21 for a 28-day	Placebo	PFS	July 2014	December 2020	Recruiting
NCT02867592	2	146	Phase 2 Trial of XL184 (Cabozantinib) an Oral Small-Molecule Inhibitor of Multiple Kinases, in Children and Young Adults with Refractory Sarcomas, Wilms Tumor, and Other Rare Tumors	Relapsing or metastatic	Rare tumors including osteosarcoma	Cabozantinib D1–28	NA	ORR	May 2017	June 2020	Recruiting
NCT04351308	2	60	A Randomized Trial of Comparison of MAPI+Camrelizumbab Verus API+Apatinib Versus MAPI in Patients with a Poor Response to Preoperative Chemotherapy for Newly Diagnosed High-grade Osteosarcomas: an Open-label, Exploratory Study	Poor Response to Preoperative Chemotherapy for Newly Diagnosed High-grade Osteosarcoma	Osteosarcoma	MAPI + Apatinib (500 mg daily) or API + Camrelizumab 200 mg every 2 weeks	MAPI	Event-free survival	May 2020	December 2022	Recruiting
